# The genome sequence of the Common Sycamore Aphid, *Drepanosiphum platanoidis *(Schrank, 1801)

**DOI:** 10.12688/wellcomeopenres.20169.1

**Published:** 2023-10-25

**Authors:** Liam M. Crowley, Reuben James, University of Oxford and Wytham Woods Genome Acquisition Lab, Darwin Tree of Life Barcoding collective, Wellcome Sanger Institute Tree of Life programme, Wellcome Sanger Institute Scientific Operations: DNA Pipelines collective, Tree of Life Core Informatics collective, Darwin Tree of Life Consortium

**Affiliations:** 1 Department of Biology, University of Oxford, Oxford, England, UK; 2 Hogenhout Lab, John Innes Centre Department of Crop Genetics, Norwich, England, UK

**Keywords:** Drepanosiphum platanoidis, common Sycamore Aphid, genome sequence, chromosomal, Hemiptera

## Abstract

We present a genome assembly from an individual female *Drepanosiphum platanoidis* (the Common Sycamore Aphid; Arthropoda; Insecta; Hemiptera; Aphididae). The genome sequence is 284.5 megabases in span. Most of the assembly is scaffolded into 15 chromosomal pseudomolecules. The mitochondrial genome has also been assembled and is 19.45 kilobases in length. Gene annotation of this assembly on Ensembl identified 13,286 protein coding genes.

## Species taxonomy

Eukaryota; Metazoa; Eumetazoa; Bilateria; Protostomia; Ecdysozoa; Panarthropoda; Arthropoda; Mandibulata; Pancrustacea; Hexapoda; Insecta; Dicondylia; Pterygota; Neoptera; Paraneoptera; Hemiptera; Sternorrhyncha; Aphidomorpha; Aphidoidea; Aphididae; Drepanosiphinae; *Drepanosiphum; Drepanosiphum platanoidis* (Schrank, 1801) (NCBI:txid527648).

## Background

The Common Sycamore Aphid, *Drepanosiphum platanoidis* (Schrank, 1801), is a holocyclic aphid, feeding and reproducing on sycamore, *Acer pseudoplatanus* L ( Wade & Leather, 2002). All adult *D. platanoidis* are winged, most are green, although a small percentage can be red, and they can develop dark cross-bars on their abdomen ( Stroyan, 1977). *D. platanoidis* is found wherever sycamore occurs, mostly in Europe and the UK, but also in the USA, Canada, New Zealand, central Asia, and North Africa ( GBIF Secretariat, 2023).

The lifecycle of *D. platanoidis* is closely linked to the nutritional value of the sycamore phloem. In the spring, the fundatrices emerge and begin asexual reproduction in response to the high nutritional value of the host phloem associated with bud burst and leaf flushing. Following this period of rapid population growth, they enter a period of aestivation of up to 8 weeks throughout the summer when conditions, such as rainfall, are less favourable ( Wellings *et al.*, 1985). In the autumn when the nutritional value increases once again, *D. platanoides* resumes feeding and reproduction. At this time, sexual male and females are produced, which subsequently mate, resulting in the laying of overwintering eggs ( Wade & Leather, 2002; Wynne *et al.*, 1994).

This species forms characteristic, uniformly spaced aggregations on the underside of sycamore leaves. The density of these aggregation depends on the palatability of the host and the associated level of intraspecific competition ( Dixon & Logan, 1972), with larger leaves supporting a greater number and higher density of aphids ( Dixon & McKay, 1970).

The reproductive rate of *D. platanoidis* is influenced by several factors including temperature, intraspecific competition and the levels of amino acids in host tissue ( Wellings, 1981). Due to this aphid’s intimate relationship with the climate in which it resides, *D. platanoidis* is a good model insect for study of the effect of climate change on insect populations ( Senior *et al.*, 2020). The availability of the genome will facilitate studies of the underlying genetics behind this aphid’s response to climate change. It will also enable genetic comparisons with other tree-dwelling and non-tree dwelling aphids.

The genome of the common sycamore aphid, *Drepanosiphum platanoidis*, was sequenced as part of the Darwin Tree of Life Project, a collaborative effort to sequence all named eukaryotic species in the Atlantic Archipelago of Britain and Ireland. Here we present a chromosomally complete genome sequence for *Drepanosiphum platanoidis*, based on one female specimen from Wytham Woods, Oxfordshire, UK.

## Genome sequence report

**Figure 1.**
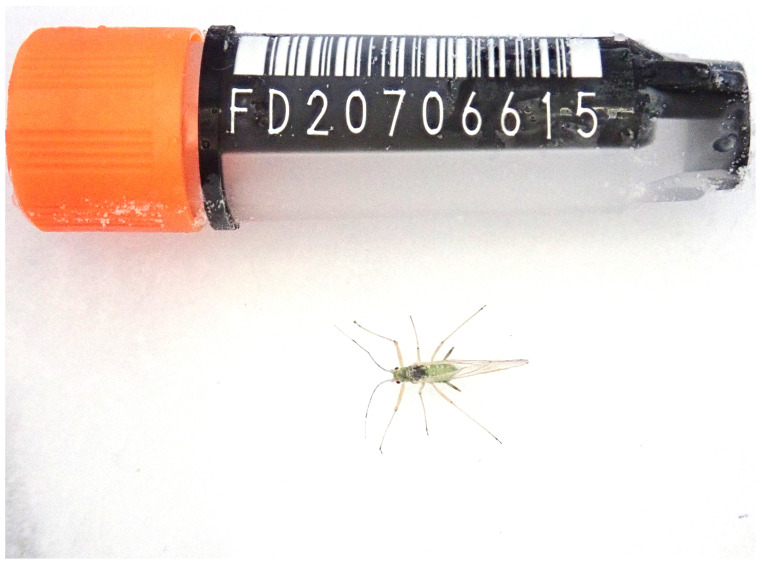
Photograph of the *Drepanosiphum platanoidis* (ihDrePlat2) specimen used for genome sequencing

The genome was sequenced from one female *Drepanosiphum platanoidis* ( Figure 1) collected from Wytham Woods, Oxfordshire, UK (51.77, –1.33). A total of 64-fold coverage in Pacific Biosciences single-molecule HiFi long reads was generated. Primary assembly contigs were scaffolded with chromosome conformation Hi-C data. Manual assembly curation corrected 94 missing joins or misjoins and removed 3 haplotypic duplications, reducing the scaffold number by 23.81% and increasing the scaffold N50 by 11.22%.

**Figure 2.**
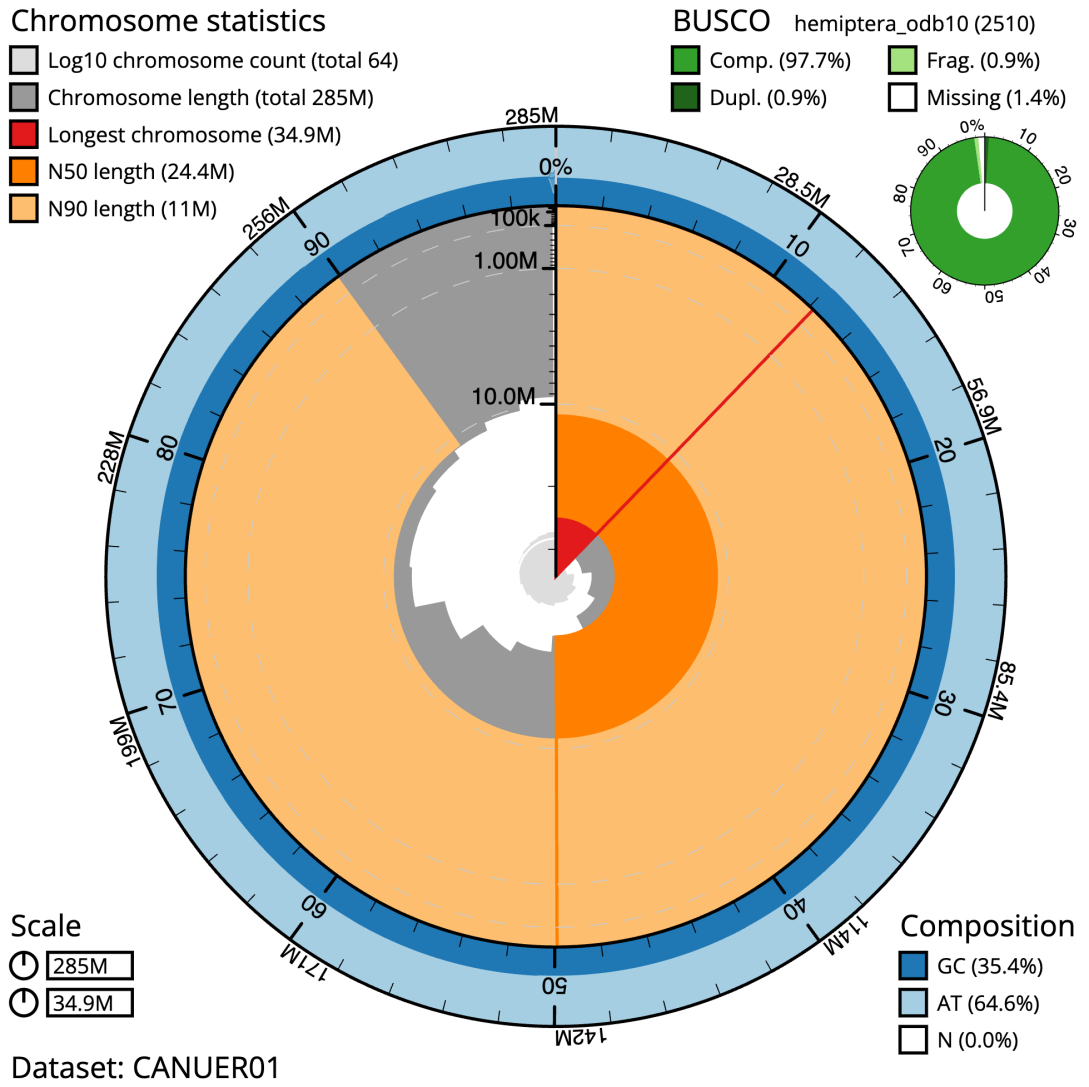
Genome assembly of *Drepanosiphum platanoidis*, ihDrePlat2.1: metrics. The BlobToolKit Snailplot shows N50 metrics and BUSCO gene completeness. The main plot is divided into 1,000 size-ordered bins around the circumference with each bin representing 0.1% of the 284,524,492 bp assembly. The distribution of scaffold lengths is shown in dark grey with the plot radius scaled to the longest scaffold present in the assembly (34,895,330 bp, shown in red). Orange and pale-orange arcs show the N50 and N90 scaffold lengths (24,435,353 and 11,045,042 bp), respectively. The pale grey spiral shows the cumulative scaffold count on a log scale with white scale lines showing successive orders of magnitude. The blue and pale-blue area around the outside of the plot shows the distribution of GC, AT and N percentages in the same bins as the inner plot. A summary of complete, fragmented, duplicated and missing BUSCO genes in the hemiptera_odb10 set is shown in the top right. An interactive version of this figure is available at https://blobtoolkit.genomehubs.org/view/Drepanosiphum/dataset/CANUER01/snail.

**Figure 3.**
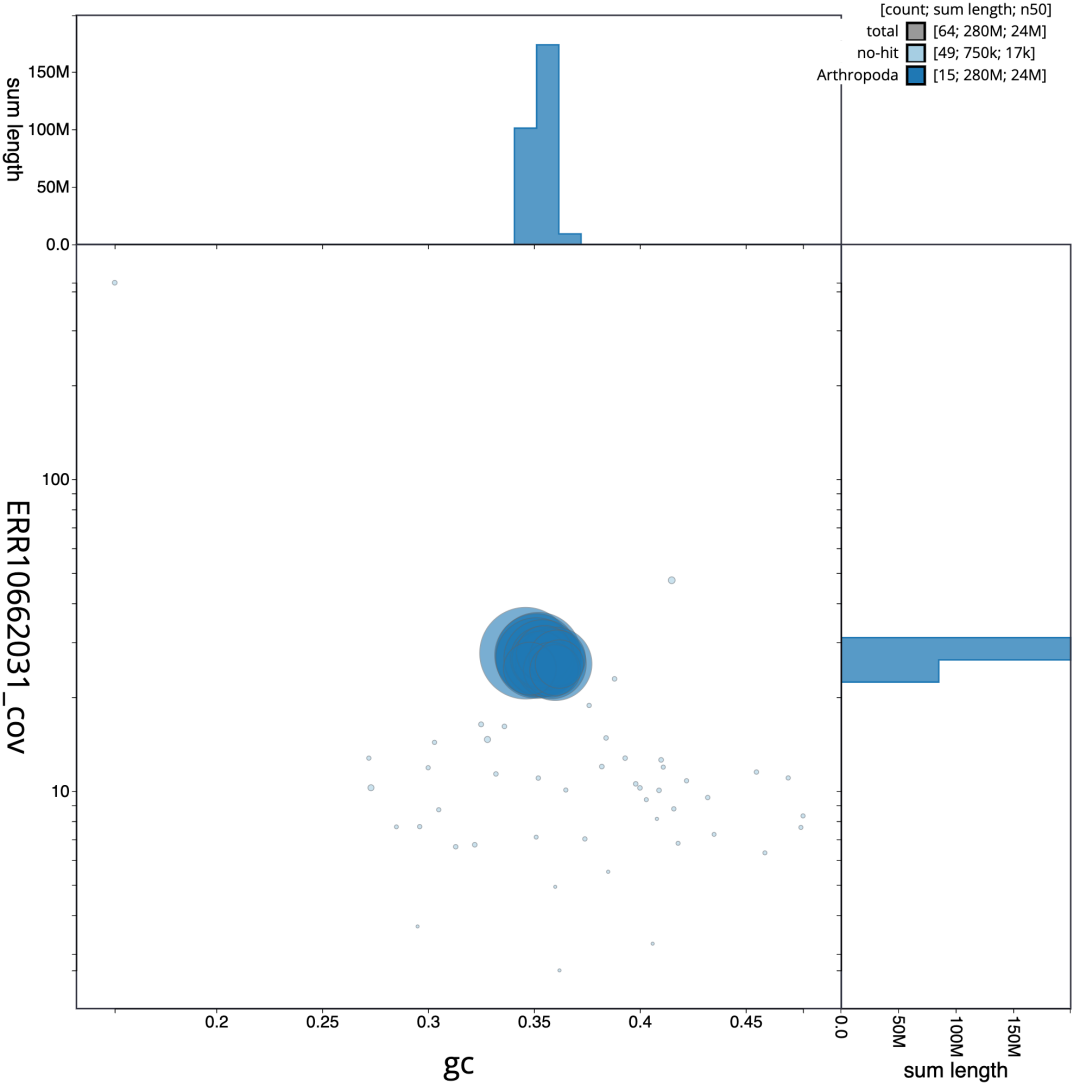
Genome assembly of *Drepanosiphum platanoidis*, ihDrePlat2.1: BlobToolKit GC-coverage plot. Scaffolds are coloured by phylum. Circles are sized in proportion to scaffold length. Histograms show the distribution of scaffold length sum along each axis. An interactive version of this figure is available at https://blobtoolkit.genomehubs.org/view/Drepanosiphum/dataset/CANUER01/blob.

**Figure 4.**
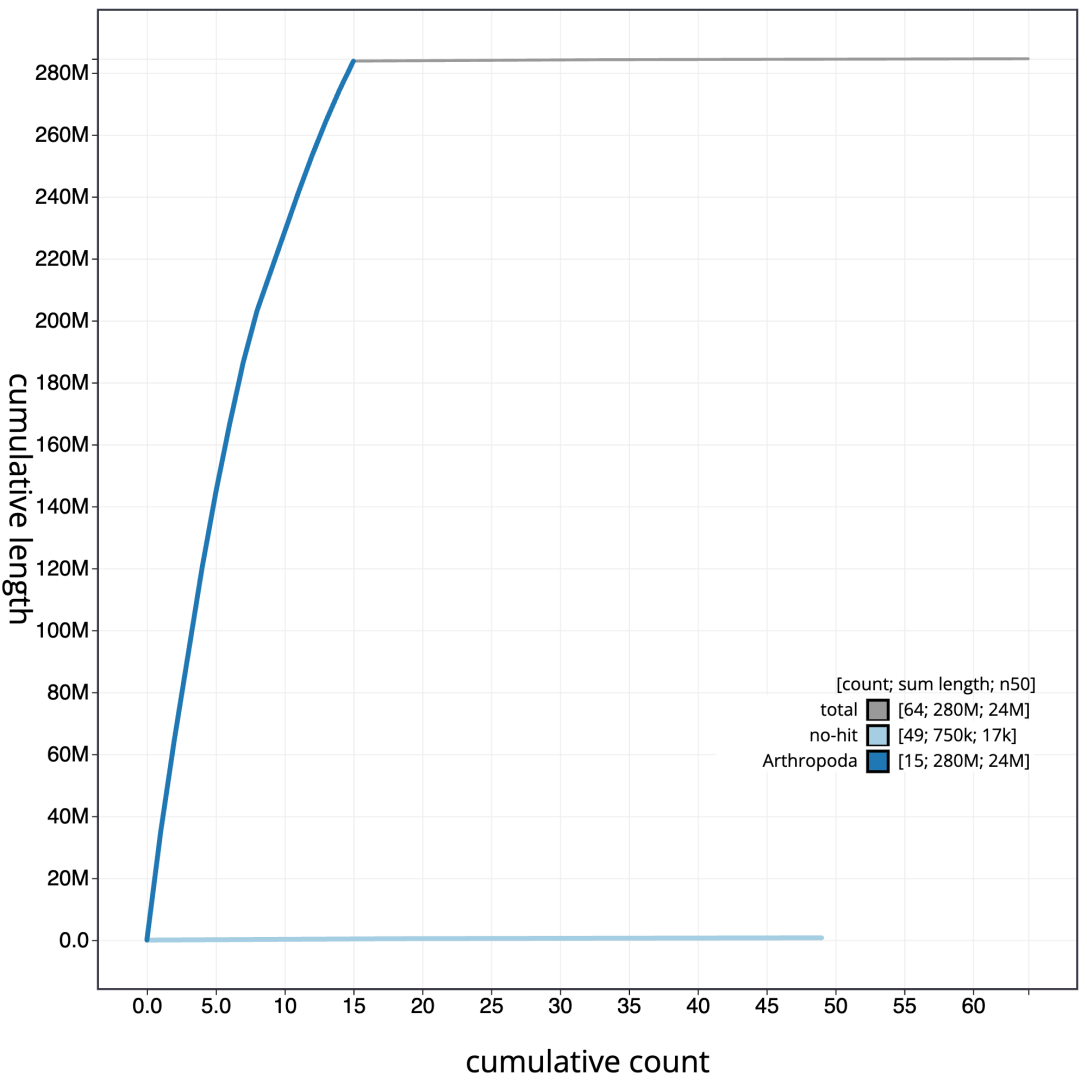
Genome assembly of *Drepanosiphum platanoidis*, ihDrePlat2.1: BlobToolKit cumulative sequence plot. The grey line shows cumulative length for all scaffolds. Coloured lines show cumulative lengths of scaffolds assigned to each phylum using the buscogenes taxrule. An interactive version of this figure is available at https://blobtoolkit.genomehubs.org/view/Drepanosiphum/dataset/CANUER01/cumulative.

**Figure 5.**
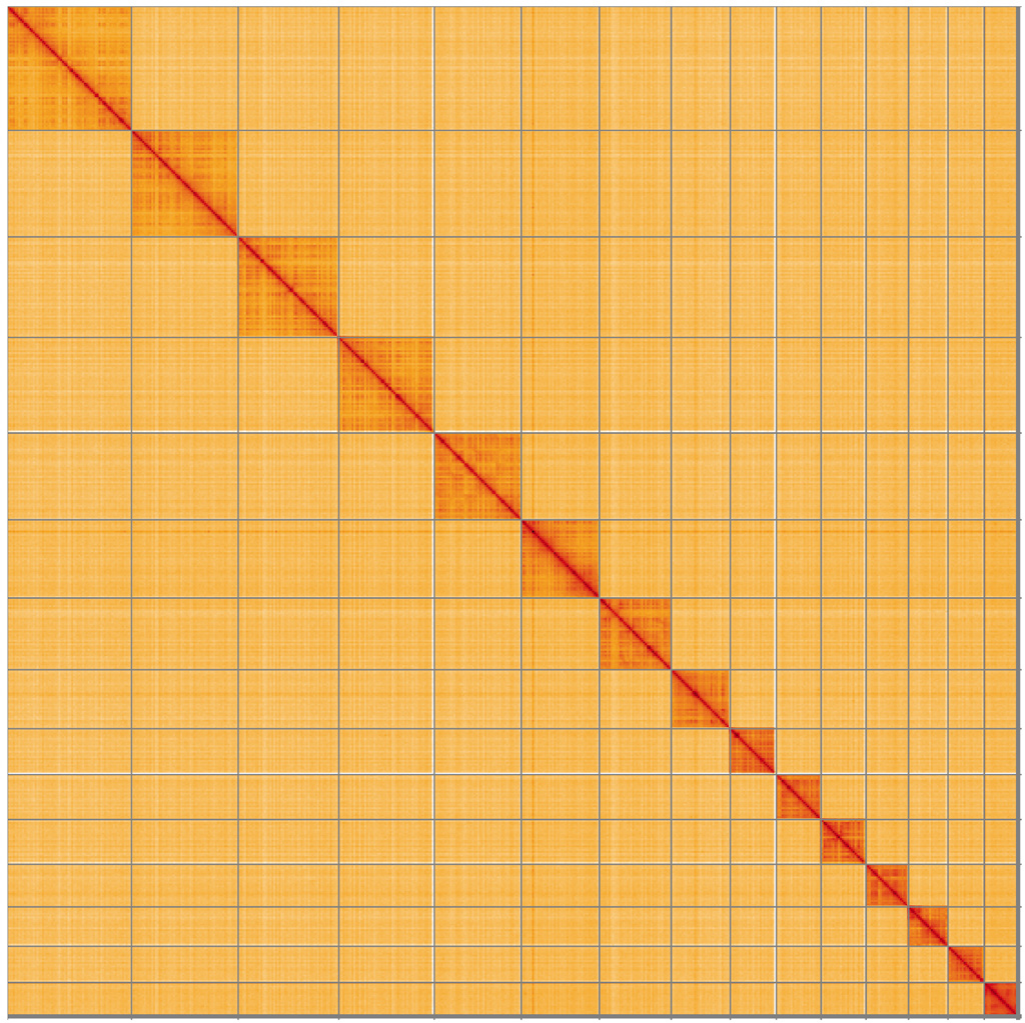
Genome assembly of *Drepanosiphum platanoidis*, ihDrePlat2.1: Hi-C contact map of the ihDrePlat2.1 assembly, visualised using HiGlass. Chromosomes are shown in order of size from left to right and top to bottom. An interactive version of this figure may be viewed at https://genome-note-higlass.tol.sanger.ac.uk/l/?d=cT_I_pNmQkC6kH1sJdMFsQ.

The final assembly has a total length of 284.5 Mb in 63 sequence scaffolds with a scaffold N50 of 24.4 Mb ( Table 1). The snailplot in Figure 2 provides a summary of the assembly statistics, while the distribution of assembly scaffolds on GC proportion and coverage is shown in Figure 3. The cumulative assembly plot in Figure 4 shows curves for subsets of scaffolds assigned to different phyla. Most (99.76%) of the assembly sequence was assigned to 15 chromosomal-level scaffolds. Chromosome-scale scaffolds confirmed by the Hi-C data are named in order of size ( Figure 5; Table 2). Three super scaffolds align to the *M. periscae* and *A. pisum* X chromosome (which is broadly conserved in aphids ( Mathers *et al.*, 2021). No male samples were available to determine which chromosome(s) are functionally the sex (X) chromosome in this species. While not fully phased, the assembly deposited is of one haplotype. Contigs corresponding to the second haplotype have also been deposited. The mitochondrial genome was also assembled and can be found as a contig within the multifasta file of the genome submission.

**Table 1.** Genome data for *Drepanosiphum platanoidis*, ihDrePlat2.1.

Project accession data		
Assembly identifier	ihDrePlat2.1	
Assembly release date	2023-01-09	
Species	*Drepanosiphum platanoidis*	
Specimen	ihDrePlat2	
NCBI taxonomy ID	527648	
BioProject	PRJEB58087	
BioSample ID	SAMEA10978756	
Isolate information	ihDrePlat2, female: whole organism (DNA sequencing) ihDrePlat1, female: whole organism (Hi-C scaffolding)	
Assembly metrics *		*Benchmark*
Consensus quality (QV)	58.4	*≥ 50*
*k*-mer completeness	100%	*≥ 95%*
BUSCO **	C:97.7%[S:96.9%,D:0.9%],F:0.9%,M:1.4%,n:2,510	*C ≥ 95%*
Percentage of assembly mapped to chromosomes	99.76%	*≥ 95%*
Sex chromosomes	Not assigned	*localised homologous* *pairs*
Organelles	Mitochondrial genome assembled	*complete single alleles*
Raw data accessions		
PacificBiosciences SEQUEL II	ERR10662031	
Hi-C Illumina	ERR10659260	
Genome assembly		
Assembly accession	GCA_948098885.1	
*Accession of alternate haplotype*	GCA_948098895.1	
Span (Mb)	284.5	
Number of contigs	751	
Contig N50 length (Mb)	0.7	
Number of scaffolds	63	
Scaffold N50 length (Mb)	24.4	
Longest scaffold (Mb)	34.9	
Genome annotation		
Number of protein-coding genes	13,286	
Number of gene transcripts	13,442	

* Assembly metric benchmarks are adapted from column VGP-2020 of “Table 1: Proposed standards and metrics for defining genome assembly quality” from ( Rhie *et al.*, 2021). ** BUSCO scores based on the hemiptera_odb10 BUSCO set using v5.3.2. C = complete [S = single copy, D = duplicated], F = fragmented, M = missing, n = number of orthologues in comparison. A full set of BUSCO scores is available at https://blobtoolkit.genomehubs.org/view/Drepanosiphum/dataset/CANUER01/busco.

**Table 2.** Chromosomal pseudomolecules in the genome assembly of *Drepanosiphum platanoidis*, ihDrePlat2.

INSDC accession	Chromosome	Length (Mb)	GC%
OX402528.1	1	34.9	34.5
OX402529.1	2	29.96	35.0
OX402530.1	3	28.31	35.0
OX402531.1	4	26.84	35.0
OX402532.1	5	24.44	35.5
OX402533.1	6	21.97	36.0
OX402534.1	7	20.2	36.0
OX402535.1	8	16.61	35.5
OX402536.1	9	12.92	35.5
OX402537.1	10	12.62	35.5
OX402538.1	11	12.59	36.0
OX402539.1	12	12.0	36.0
OX402540.1	13	11.05	35.0
OX402541.1	14	10.19	36.0
OX402542.1	15	9.2	36.0
OX402543.1	MT	0.02	15.5

The estimated Quality Value (QV) of the final assembly is 58.4 with *k*-mer completeness of 100%, and the assembly has a BUSCO v5.3.2 completeness of 97.7% (single = 96.9%, duplicated = 0.9%), using the hemiptera_odb10 reference set ( *n* = 2,510).

Metadata for specimens, spectral estimates, sequencing runs, contaminants and pre-curation assembly statistics can be found at https://links.tol.sanger.ac.uk/species/527648.

## Genome annotation report

The *Drepanosiphum platanoidis* genome assembly (GCA_948098885.1) was annotated using the Ensembl rapid annotation pipeline ( Table 1; https://rapid.ensembl.org/Drepanosiphum_platanoidis_GCA_948098885.1/Info/Index). The resulting annotation includes 13,442 transcribed mRNAs from 13,286 protein-coding genes.

## Methods

### Sample acquisition and nucleic acid extraction

A female *Drepanosiphum platanoidis* (specimen ID Ox001587, individual ihDrePlat2) was collected from Wytham Woods, Oxfordshire (biological vice-county Berkshire), UK (latitude 51.77, longitude –1.33) on 2021-07-14. The specimen was collected and identified by Liam Crowley (University of Oxford) and preserved on dry ice.

The sample was prepared for DNA extraction at the Tree of Life laboratory, Wellcome Sanger Institute (WSI). The ihDrePlat2 sample was weighed and dissected on dry ice with tissue set aside for Hi-C sequencing. Tissue from the whole organism was disrupted using a Nippi Powermasher fitted with a BioMasher pestle. DNA was extracted at the WSI Scientific Operations core using the Qiagen MagAttract HMW DNA kit, according to the manufacturer’s instructions.

### Sequencing

Pacific Biosciences HiFi circular consensus DNA sequencing libraries were constructed according to the manufacturers’ instructions. DNA sequencing was performed by the Scientific Operations core at the WSI on the Pacific Biosciences SEQUEL II (HiFi) instrument. Hi-C data were also generated from tissue of ihDrePlat1 using the Arima2 kit and sequenced on the Illumina NovaSeq 6000 instrument.

### Genome assembly, curation and evaluation

Assembly was carried out with Hifiasm ( Cheng *et al.*, 2021) and haplotypic duplication was identified and removed with purge_dups ( Guan *et al.*, 2020). The assembly was then scaffolded with Hi-C data ( Rao *et al.*, 2014) using YaHS ( Zhou *et al.*, 2023). The assembly was checked for contamination and corrected as described previously ( Howe *et al.*, 2021). Manual curation was performed using HiGlass ( Kerpedjiev *et al.*, 2018) and Pretext ( Harry, 2022). The mitochondrial genome was assembled using MitoHiFi ( Uliano-Silva *et al.*, 2022), which runs MitoFinder ( Allio *et al.*, 2020) or MITOS ( Bernt *et al.*, 2013) and uses these annotations to select the final mitochondrial contig and to ensure the general quality of the sequence.

A Hi-C map for the final assembly was produced using bwa-mem2 ( Vasimuddin *et al.*, 2019) in the Cooler file format ( Abdennur & Mirny, 2020). To assess the assembly metrics, the *k*-mer completeness and QV consensus quality values were calculated in Merqury ( Rhie *et al.*, 2020). This work was done using Nextflow ( Di Tommaso *et al.*, 2017) DSL2 pipelines “sanger-tol/readmapping” ( Surana *et al.*, 2023a) and “sanger-tol/genomenote” ( Surana *et al.*, 2023b). The genome was analysed within the BlobToolKit environment ( Challis *et al.*, 2020) and BUSCO scores ( Manni *et al.*, 2021; Simão *et al.*, 2015) were calculated.

Table 3 contains a list of relevant software tool versions and sources.

**Table 3.** Software tools: versions and sources.

Software tool	Version	Source
BlobToolKit	4.0.7	https://github.com/blobtoolkit/blobtoolkit
BUSCO	5.3.2	https://gitlab.com/ezlab/busco
Hifiasm	0.16.1-r375	https://github.com/chhylp123/hifiasm
HiGlass	1.11.6	https://github.com/higlass/higlass
Merqury	MerquryFK	https://github.com/thegenemyers/MERQURY.FK
MitoHiFi	2	https://github.com/marcelauliano/MitoHiFi
PretextView	0.2	https://github.com/wtsi-hpag/PretextView
purge_dups	1.2.3	https://github.com/dfguan/purge_dups
sanger-tol/ genomenote	v1.0	https://github.com/sanger-tol/genomenote
sanger-tol/ readmapping	1.1.0	https://github.com/sanger-tol/readmapping/tree/1.1.0
YaHS	1.2a	https://github.com/c-zhou/yahs

### Genome annotation

The BRAKER2 pipeline ( Brůna *et al.*, 2021) was used in the default protein mode to generate annotation for the *Drepanosiphum platanoidis* assembly (GCA_948098885.1) in Ensembl Rapid Release.

### Wellcome Sanger Institute – Legal and Governance

The materials that have contributed to this genome note have been supplied by a Darwin Tree of Life Partner. The submission of materials by a Darwin Tree of Life Partner is subject to the **‘Darwin Tree of Life Project Sampling Code of Practice’,** which can be found in full on the Darwin Tree of Life website here. By agreeing with and signing up to the Sampling Code of Practice, the Darwin Tree of Life Partner agrees they will meet the legal and ethical requirements and standards set out within this document in respect of all samples acquired for, and supplied to, the Darwin Tree of Life Project.

Further, the Wellcome Sanger Institute employs a process whereby due diligence is carried out proportionate to the nature of the materials themselves, and the circumstances under which they have been/are to be collected and provided for use. The purpose of this is to address and mitigate any potential legal and/or ethical implications of receipt and use of the materials as part of the research project, and to ensure that in doing so we align with best practice wherever possible. The overarching areas of consideration are:

•   Ethical review of provenance and sourcing of the material

•   Legality of collection, transfer and use (national and international)

Each transfer of samples is further undertaken according to a Research Collaboration Agreement or Material Transfer Agreement entered into by the Darwin Tree of Life Partner, Genome Research Limited (operating as the Wellcome Sanger Institute), and in some circumstances other Darwin Tree of Life collaborators.

## Data Availability

European Nucleotide Archive: *Drepanosiphum platanoidis* (common sycamore aphid). Accession number PRJEB58087; https://identifiers.org/ena.embl/PRJEB58087. ( Wellcome Sanger Institute, 2023) The genome sequence is released openly for reuse. The *Drepanosiphum platanoidis* genome sequencing initiative is part of the Darwin Tree of Life (DToL) project. All raw sequence data and the assembly have been deposited in INSDC databases. Raw data and assembly accession identifiers are reported in Table 1.

## Author information

Members of the University of Oxford and Wytham Woods Genome Acquisition Lab are listed here: https://doi.org/10.5281/zenodo.7125292.

Members of the Darwin Tree of Life Barcoding collective are listed here: https://doi.org/10.5281/zenodo.4893703.

Members of the Wellcome Sanger Institute Tree of Life programme are listed here: https://doi.org/10.5281/zenodo.4783585.

Members of Wellcome Sanger Institute Scientific Operations: DNA Pipelines collective are listed here: https://doi.org/10.5281/zenodo.4790455.

Members of the Tree of Life Core Informatics collective are listed here: https://doi.org/10.5281/zenodo.5013541.

Members of the Darwin Tree of Life Consortium are listed here: https://doi.org/10.5281/zenodo.4783558.
