# Supplementary material for: The genome sequence of the Common Sycamore Aphid, Drepanosiphum platanoidis (Schrank, 1801)
Source: Wellcome Open Res. 2023 Oct 25;8:481. [Version 1] doi: 10.12688/wellcomeopenres.20169.1 (PMC11347910; doi:10.12688/wellcomeopenres.20169.1)
Supplement: Peer review history

# Reviewer report: Reviewer response for version 1

**DOI:** 10.21956/wellcomeopenres.22332.r81065

**Published:** 2024-08-26

**Authors:** Maria Sharakhova

**Affiliations:** 1 Virginia Polytechnic and State University, Blacksburg, USA

The manuscript by Crowley L.M. with co-authors "The genome sequence of common sycamore aphid *Drepanosiphum platanoidis*" is a brief report of a de novo sequenced genome of an aphid that feeds and reproduces on sycamore plants. The genome was generated from a single female using the most efficient modern sequencing technology Pacific Biosciences (HiFi) and assembled to the chromosome level using the Hi-C scaffolding approach. The genome assembly is 284.5 Mb in size and consists of 15 chromosomal pseudomolecules. The sex chromosome was not determined as only female sample was sequenced. The mitochondrial genome has also been assembled. The genome was sequenced as part of the Darwin Tree of Life Project, which aims to provide sequencing data for all named eukaryotic species in the UK and Ireland.

**Major concerns:**

 1.   In the introductory section, please explain specific terminology, such as the meaning of a holocyclic aphid.

 2.   Although this manuscript is a short report of the de novo genome assembly, I think a small discussion of the results would be very useful here. For example, the discussion can briefly include a comparison of the genome quality with other genomes sequenced under the Darwin Tree of Life project, and a comparison with genetic data previously obtained for this species, such as chromosome number, genome size estimate, and so on. I think this information would be very useful for the readers and can stimulate further research on the genetics of this species.

 3.    If this tube is shown to provide a scale for the size of the sample, this needs to be explained in the figure legend. However, it is better to use a ruler with a scale in centimeters. I would suggest reformatting this figure to include a scale in centimeters.

 4.   I would also suggest numbering the scaffolds and adding a scale in Mb to indicate the sizes of the chromosome scaffolds in Figure 5. It would also be interesting if you could indicate which chromosomes are related to the X chromosome in closely related insects.

**Minor concern:**

 Please, correct this typo:

 Three super scaffolds align to the *M. periscae* and *A. pisum* X chromosome (which is broadly

 conserved in aphids -> Three super scaffolds align to the *M. periscae* and *A. pisum* X chromosome, which is broadly conserved in aphids

Are sufficient details of methods and materials provided to allow replication by others?

Yes

Is the rationale for creating the dataset(s) clearly described?

Yes

Are the datasets clearly presented in a useable and accessible format?

Yes

Are the protocols appropriate and is the work technically sound?

Yes

Reviewer Expertise:

mosquito genetics and genomics

I confirm that I have read this submission and believe that I have an appropriate level of expertise to confirm that it is of an acceptable scientific standard.

# Reviewer report: Reviewer response for version 1

**DOI:** 10.21956/wellcomeopenres.22332.r81060

**Published:** 2024-05-20

**Authors:** Felipe Cordeiro Dias

**Affiliations:** 1 Universidade de São Paulo, São Paulo, Brazil

The article provides a complete description of the assembly and annotation of the genome of the common sycamore aphid *Drepanosiphum platanoidis,* detailing aspects of its composition and structure. The report is well written and provides a good overview.

 The background shows some information about the biological aspects of the species, such as the differences brought by the seasons and how this impacts the species and how it would be useful to understand more about the effects of climate change on natural environments. It could include more details about any genetic information that the species already possesses, if applicable, or even about the group in which the species is located.

 The methods, including pipelines and programs used, and their results are clear and well presented, following the standard of most genomic notes, including information about the sources and location of raw and processed data. It would be interesting to analyze in the future how using RNASeq data together with protein data would or would not improve genome annotation, considering that here they are only using BRAKER2, adapted only for protein data.

 In short, the article is well written and developed in its purposes and presents a good overview of the genome.

Are sufficient details of methods and materials provided to allow replication by others?

Yes

Is the rationale for creating the dataset(s) clearly described?

Yes

Are the datasets clearly presented in a useable and accessible format?

Yes

Are the protocols appropriate and is the work technically sound?

Yes

Reviewer Expertise:

genetics, entomology, molecular biology, bioinformatics, evolution and phylogenetics

I confirm that I have read this submission and believe that I have an appropriate level of expertise to confirm that it is of an acceptable scientific standard.
